# Characterization of recovered walking patterns and motor control after contusive spinal cord injury in rats

**DOI:** 10.1002/brb3.71

**Published:** 2012-07-10

**Authors:** Christopher N Hansen, William Linklater, Raquel Santiago, Lesley C Fisher, Stephanie Moran, John A Buford, D Michele Basso

**Affiliations:** 1 Neuroscience Graduate Studies Program, The Ohio State UniversityColumbus, Ohio; 2 School of Health and Rehabilitation Sciences, The Ohio State UniversityColumbus, Ohio; 3 Center for Brain and Spinal Cord Repair (CBSCR), The Ohio State UniversityColumbus, Ohio

**Keywords:** Kinematics, locomotion, rehabilitation, spinal cord injury

## Abstract

Currently, complete recovery is unattainable for most individuals with spinal cord injury (SCI). Instead, recovery is typically accompanied by persistent sensory and motor deficits. Restoration of preinjury function will likely depend on improving plasticity and integration of these impaired systems. Eccentric muscle actions require precise integration of sensorimotor signals and are predominant during the yield (E2) phase of locomotion. Motor neuron activation and control during eccentric contractions is impaired across a number of central nervous system (CNS) disorders, but remains unexamined after SCI. Therefore, we characterized locomotor recovery after contusive SCI using hindlimb (HL) kinematics and electromyographic (EMG) recordings with specific consideration of eccentric phases of treadmill (TM) walking. Deficits in E2 and a caudal shift of locomotor subphases persisted throughout the 3-week recovery period. EMG records showed notable deficits in the semitendinosus (ST) during yield. Unlike other HL muscles, recruitment of ST changed with recovery. At 7 days, the typical dual-burst pattern of ST was lost and the second burst (ST2) was indistinct. By 21 days, the dual-burst pattern returned, but latencies remained impaired. We show that ST2 burst duration is highly predictive of open field Basso, Beattie, Bresnahan (BBB) scores. Moreover, we found that simple changes in locomotor specificity which enhance eccentric actions result in new motor patterns after SCI. Our findings identify a caudal shift in stepping kinematics, irregularities in E2, and aberrant ST2 bursting as markers of incomplete recovery. These residual impairments may provide opportunities for targeted rehabilitation.

## Introduction

Spinal cord injury (SCI) results in a diverse range of behavioral outcomes that depend on the type, severity, and level of injury. To date, the extent of recovered central nervous system (CNS) control over locomotion has been best elucidated in reductionistic lesion models (Kaegi et al. 2002; Ballermann et al. 2006; Johnson et al. 2012). Surprisingly, less is understood about recovery from contusion-type lesions, which replicate human SCI. Contusive SCI results in complex pathology with distinct anatomical, behavioral, and cellular sequella along the neuraxis (Stokes and Jakeman 2002; Profyris et al. 2004; Detloff et al. 2008). It is well-accepted that greater sparing of descending midbrain/brainstem pathways improve motor function after contusion (Fehlings and Tator 1995; Basso et al. 2002; Schucht et al. 2002). However, factors that promote supraspinal and afferent integration during locomotion have received little attention.

Differential recovery after contusive SCI may be identified by changes in gait biomechanics and muscle activation patterns. After hemisection, postural elevation, interlimb uncoupling, and aberrant coactivation patterns between adjacent muscles persist and indicate the limits of recovery (Kaegi et al. 2002; Ballermann et al. 2006). Given the compensatory nature of this injury, it is unclear whether similar factors delineate recovery after bilateral contusion. We previously identified at least one motor feature that remains impaired after SCI – the yield phase during weight acceptance (Basso et al. 1994). Here, we ask whether the kinematics or electromyographic (EMG) metrics of yield may be associated with the extent of recovery.

Eccentric motor control represents a hallmark of skilled locomotion that is impaired across CNS injury models, but remains unexamined after contusive SCI (Helgren and Goldberger 1993; Basso et al. 1994; Damiano et al. 2001; Dibble et al. 2009). In this ubiquitous action, motor units are partially recruited to keep muscle force below the external load. To attain effective eccentric muscle lengthening, descending drive is precisely controlled to match the afferent input of the movement (Enoka 1996). A predominant eccentric period in the step cycle occurs prior to ground contact and during weight acceptance, when hamstring muscles like the semitendinosus (ST) lengthen to decelerate the hindlimb (HL) and dissipate impact forces during yield (E2). Importantly, recruitment of ST adapts to a variety of locomotor conditions and requires descending control for optimal function (Buford et al. 1990; Pratt et al. 1996; Smith et al. 1998). Our previous work in the cat shows that the eccentric phase of locomotion remains impaired despite marked recovery from a hemisection (Basso et al. 1994). To further this observation and identify mechanisms of eccentric control after contusion, we examined ST recruitment patterns over time and at recovery plateau. Whether poor eccentric activity in ST or other HL muscles prevents optimal recovery is unknown.

The present study was designed to identify features of recovered walking patterns that differentiate functional restitution after a mild/moderate, midthoracic contusion injury. Detailed assessment of HL muscle recruitment and joint kinematics describe the extent of motor control. Our findings suggest that eccentric actions of ST provide novel insight into mechanisms of locomotor recovery after SCI.

## Materials and Methods

### Subjects and surgeries

Experiments were conducted in 14 female Sprague-Dawley rats (250–300 g, Harlan, Indianapolis, Indiana) that were randomly assigned to control laminectomy (LAM) or SCI groups following EMG implantation. Naive data collection for all rats served as baseline. Comparisons included Naive (*n* = 14), LAM (*n* = 5), and SCI (*n* = 9). Animals were housed 2–3 per cage in a controlled environment (12 h light/dark cycle) with food and water available ad libitum. Housing, surgical procedures, and assessment of behavior was done in accordance with The Ohio State University Laboratory Animal Care and Use Committee. For all surgeries, rats were anesthetized intraperitoneal (i.p.) with ketamine (80 mg/kg) and xylazine (20 mg/kg). During each surgical procedure, a heating pad maintained body temperature. Prophylactic antibiotics (gentomycin sulfate 1 mg/kg) and saline were given post surgery to prevent infection and dehydration.

#### EMG implantation

Subjects were acclimated to the treadmill (TM) and trained to walk steadily prior to EMG implantation; this training required 2–3 weeks. During the first surgery, bipolar EMG electrodes were implanted into the tibialis anterior (TA), lateral gastrocnemius (LG), and the ST of the left HL. These muscles were selected based on electrode stability and locomotor biomechanics. A small rostral–caudal incision was made along the sagittal suture of the skull and four screws were anchored on each side. Teflon-coated, multistranded stainless steel wires fixed to a head plug were routed subcutaneously to the HL and implanted into exposed muscles with a hypodermic needle. Electrode functionality was confirmed by electrical stimulation through each lead (∼0.2–0.8 mA, 0.2 msec cathodal pulse) to elicit a muscle twitch. A ground electrode remained subcutaneous to serve as reference. The head connector was cemented with varnish and dental acrylic to the screws, and incisions were closed with suture.

#### Spinal cord injury

In the time between EMG implant and SCI (11 days), normal open field locomotion was confirmed for each rat. Over this period, rats were reacclimated to the TM and learned to walk with the EMG wire connected to the head plug. This data collection was used for naive comparison. In the second surgery, rats were anesthetized as described previously, and a midthoracic T8 laminectomy exposed the spinal cord. Animals randomized to the SCI group received a mild/moderate injury produced by rapidly impacting the spinal cord using the OSU Electromagnetic Spinal Cord Injury Device or the Infinite Horizons (IH) Device (Stokes et al. 1992; Stokes and Jakeman 2002). Following contusion or LAM control, dorsal musculature was sutured and skin was closed using surgical clips. Sterile saline was administered subcutaneously to prevent dehydration. Antibiotics were delivered daily and bladders were manually expressed 2×/day until the bladder reflex returned. Vitamin C pellets were given to prevent urinary tract infections (Behrmann et al. 1992). Animals that exhibited wiring problems or bladder infection following surgery were not used for EMG collection (*n* = 2).

### EMG recording

To examine muscle recruitment patterns after SCI, EMG signals were recorded and synchronized with joint kinematics for six animals and averaged across at least 20 steps on the TM (Columbus Instruments, Columbus, Ohio). For downhill recordings, the TM belt was set to a 10% (5.7 degrees) downslope grade. Flexible insulated cables were attached to a head plug, and connected via a commutator to the amplifier, allowing free movement of the subjects on the TM belt. A sugar water dispenser at the front of the belt prompted forward locomotion. Preoperative training frequency and duration was adjusted per rat until long bouts of sustained stepping occurred while drinking. Postoperatively, brief exposure to the TM occurred to maintain comfort with the task. Collection occurred at the same speed (12 m/min) and while drinking to eliminate backward drift.

The EMG signals were amplified at a gain of 1K with an AM-Systems model 1700 differential amplifier. The bandpass filters were set for 20 Hz–5 KHz, and a 60-Hz notch filter was engaged. Computerized data acquisition was accomplished with a sampling rate of 2 KHz using either a 16-bit Datapac 2K2 system (Run Technologies, Laguna Hills, California) or a 16-bit CED Power 1401 system with Spike2 software (CED, Cambridge, UK). The EMG records were adjusted to remove DC offsets, rectified, and averaged across 20+ steps off-line using a custom script that used initial contact times as a triggering event. A burst detection program determined the beginning (onset) and end (offset) of each EMG burst and calculated relative to initial contact by determining when the EMG level crossed a threshold set to 2 standard deviations above the mean activity level during quiescence for each muscle. Visual inspection was used to adjust onset and offset times as required to eliminate spurious bursts and locate the main burst periods associated with locomotion. Burst durations were calculated based on the onset and offset times. Digital video records were synchronized with the EMG recordings by means of an LED light that was visible to the camera, with the voltage pulse for the light recorded along with the EMG.

### Locomotor assessments

Locomotor recovery was assessed using the 21-point Basso, Beattie, Bresnahan (BBB) locomotor rating scale (Basso et al. 1995). Scores range from no HL movement (0) to normal locomotor function (21). Rating criteria considered joint movement, weight support, plantar stepping, coordination, toe clearance, paw position, as well as trunk and tail control. Open field activity of each rat occurred for 4 min by two raters blind to group assignment. Assessments were done prior to injury, at 1 and 7 days postoperatively (dpo), and weekly thereafter.

#### Two-dimensional kinematics

All rats had two-dimensional (2D) kinematic recordings of TM walking before and 3 weeks after SCI. Left HLs were shaved and bony prominences were marked with permanent marker preoperatively. The prominences included the iliac crest, greater trochanter, femoral condyle, lateral malleolus, and head of the fifth metatarsal. A videotape record of quadrupedal locomotion (10–20 step bouts) was collected using a Panasonic WV-CL350 camera (60 Hz) with a time-code generator. The same LED light used to synchronize the EMG and digital video records was visible to the analogue video camera and was used to synchronize the records. HL kinematic markers were digitized using PEAK Motus. To account for movement of the knee joint, a triangulation program was used to estimate its position (Goslow et al. 1973). Actual femur and tibia bone lengths were collected at sacrifice and used with the hip and ankle X, Y positions to derive location. Angular excursions were calculated for the hip, knee, and ankle during each phase of quadruped gait: Initial Contact (E1), Yield (E2), Lift Off (E3), and Peak Flexion (F) (Basso et al. 1994). Timing of initial contact along with the LED synchronization light served as the reference times to synchronize EMG and kinematic data. Angle–angle diagrams were constructed by plotting joint excursions (hip–knee or knee–ankle) against one another to assess intralimb coordination.

### Histology

Rats were perfused with 0.1 M phosphate buffered saline followed by 4% paraformaldehyde. Tissue was collected and cryoprotected in sucrose. The lesion site was transversely sectioned (20 μm) and stained for myelin using eriochrome cyanine. The section with the largest lesion and least amount of stained white matter represented the lesion epicenter. Area of stained white matter at the epicenter was divided by the total cross-sectional area of an uninjured cord at the same vertebral level to serve as a measure of injury severity (Kloos et al. 2005).

### Statistics

All outcome measures were analyzed compared to naive. Kinematic comparisons were done using a repeated measures analysis of variance (ANOVA) and Tukey's post hoc test. Significance observed with BBB scores was determined using a Mann–Whitney *U*-test to account for unequal sample size. Correlations between EMG burst duration, BBB score, and white matter sparing were done using Pearson's correlation analysis. Significance was set at *P* < 0.05 and mean ± SEM are shown.

## Results

### Residual deficits contribute to a new walking strategy after mild SCI

**Figure 1:**
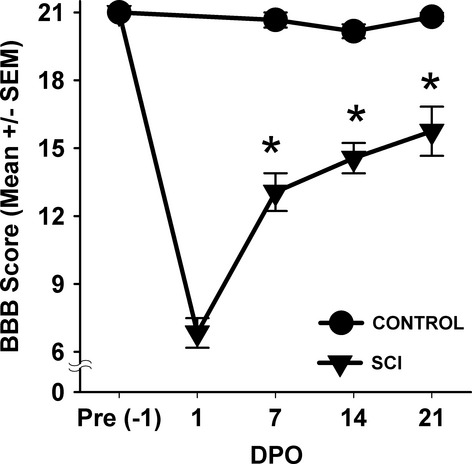
Open field locomotion. Spontaneous recovery occurred in the open field after mild SCI. BBB scores plateaued by 21 days and remained significantly lower than control (mean SCI = 15.7 ± 1.085). Residual deficits at 21 days included toe dragging, paw rotation, and trunk instability. Note that controls showed a nonsignificant reduction in performance at 14 days due to mild trunk instability. Data are reported as mean ± standard error of the mean (significance determined by a Mann–Whitney *U-*test; *P* < 0.05).

Using the BBB scale, spontaneous recovery occurred over 21 days after mild SCI but residual impairments prevented normal locomotion (Fig. 1). Mild SCI resulted in severe paresis with slight and extensive HL movements 1 day after SCI (Mean BBB = 6.83 ± 0.655). Weight supported stepping recovered within 7 days. Despite rapid improvement, recovery plateaued at levels significantly below normal at 21 days (Mean BBB = 15.75 ± 1.085; *P* < 0.05). While one animal attained near normal locomotion (BBB = 19), remaining animals had persistent trunk instability (100%), toe dragging (37.5%), and paw rotation at lift off (100%) or initial contact (37.5%).

**Figure 2:**
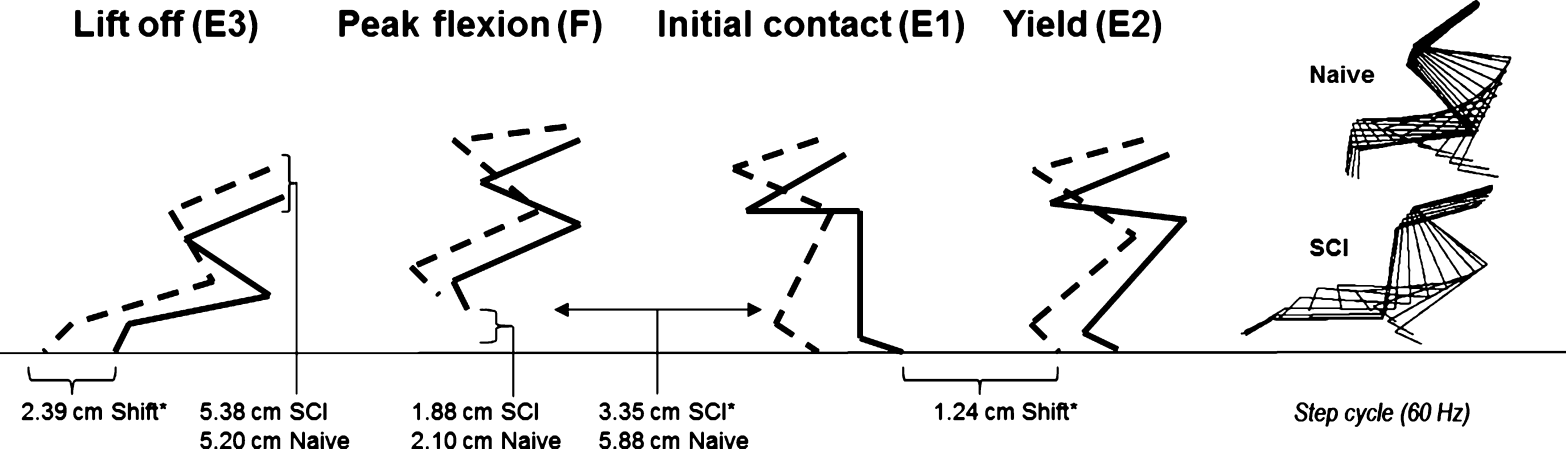
Stick figure diagrams at the end of each phase of gait illustrate prolonged extension during TM locomotion. Subphases of locomotion include E1, E2, E3, and F. The third extension phase (E3) occurs from midstance to lift off where knee and ankle extension is greatest. The flexion phase (F) runs from lift off to maximum knee and ankle flexion. The first extension phase (E1) occurs from peak flexion to initial contact. Weight acceptance of the limb results in flexion of the knee and ankle called yield (E2). A caudal shift is evident during all phases of locomotion after SCI (dotted line) compared to normal (solid line). On average, lift off occurs 2.39 ± 0.23 cm and initial contact occurs 1.24 ± 0.29 cm more caudal than normal (*P* < 0.01). Lower toe height occurred at peak flexion (1.88 ± 0.151 cm, SCI; 2.10 ± 0.174 cm, Naive) and decreased limb advancement during E1 (3.35 ± 0.473 cm, SCI; 5.88 ± 0.488 cm, Naive; *P* < 0.05) indicate hypometria during swing. An elevated crest was observed in 60% of animals (5.38 ± 0.29 cm, SCI; 5.20 ± 0.14 cm, Naive).

Using 2D TM kinematics, we quantified the plateaued walking behavior across subphases of locomotion (Fig. 2; Basso et al. 1994). Hip movements are biphasic and include two subphases, flexion (F) and extension (E). Knee and ankle movements are more complex and are divided into four subphases (E1, E2, E3, F). The first extension subphase (E1) occurs from peak flexion in swing until initial paw contact on the ground. The E2 subphase, from initial contact through weight acceptance, represents joint flexion during yield and relies on eccentric muscle lengthening. During E3, midstance to lift off, all joints extend. Lift off to peak flexion represents the flexion (F) subphase. Thus, stance includes E2 and E3 and swing includes F and E1 (Fig. 2).

**Figure 3:**
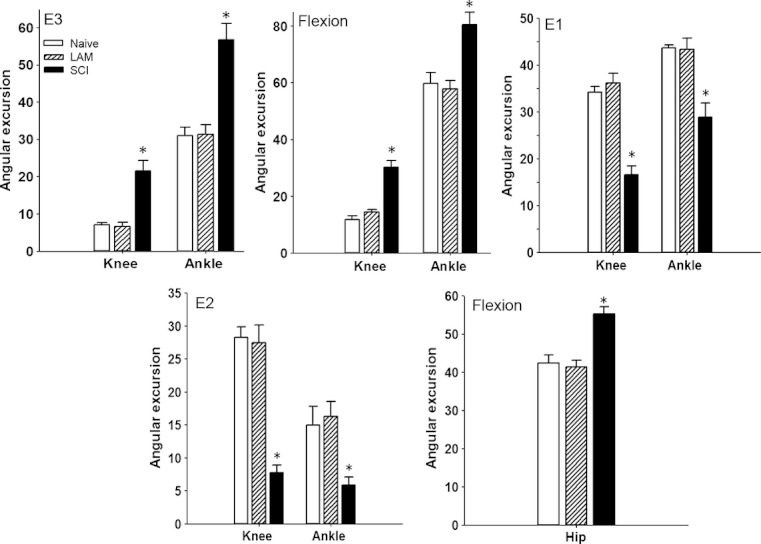
Angular excursion profiles of hip, knee, and ankle joints. Precise kinematic analysis of joint excursion between different phases of gait reveal altered biomechanics after SCI. Extension of the knee and ankle significantly increased from late stance to midswing (E2–E3, E3–F) and decreased from midswing to midstance (F–E1, E1–E2) during TM locomotion. Hip extension increased relative to naive and control. (Significance determined by repeated measures ANOVA and Tukey's post hoc test; *P* < 0.05.)

After recovery from SCI, the position of the paw relative to the pelvis showed significant caudal displacement during all phases of gait (Fig. 2). The caudal shift for injured rats (dotted lines) was 2.39 ± 0.23 cm (*P* < 0.01) at lift off and 1.24 ± 0.29 cm (*P* < 0.01) at initial contact compared to naive (solid lines; Fig. 2). During E1, a 43% reduction in forward swing occurred after SCI (3.35 ± 0.473 cm, SCI; 5.88 ± 0.488 cm, Naive; *P* < 0.05). This caudal shift was reflected in significant differences in angular excursion of all HL joints (Fig. 3). Knee and ankle extension decreased during late swing (E1) and yield (E2) (*P* < 0.05). Significantly greater extension occurred in the hip, knee, and ankle during late stance (E3), leading to more excursion during flexion (F) after SCI (*P* < 0.05). The increase in flexion was not due to hypermetria as toe height was reduced after injury (toe height: 1.88 ± 0.151 cm, SCI; 2.10 ± 0.174 cm, Naive; Fig. 2); rather, greater flexion represented the return from prolonged extension at lift off. At lift off, the pelvis was on average 0.78 cm higher after SCI in 60% of animals. Implantation of EMG electrodes did not affect joint angular excursion (compare Naive and LAM groups, Fig. 3).

### Recovery of intralimb coordination occurs in a proximal to distal manner

**Figure 4:**
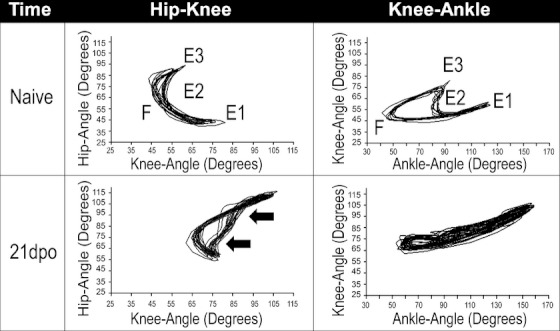
Fractionated movement in proximal and distal joints. Angle–angle plots were used to describe intralimb coordination between proximal (hip–knee) or distal (knee–ankle) joints. Naive plots depict curvilinear patterns between the hip–knee and knee–ankle to indicate normal locomotion. After recovery from SCI, coordination between distal joints is most impaired, as knee–ankle plots depict a linear rather than curvilinear pattern. Hip–knee coordination is less impaired, but shows changes in shape and position. The most obvious change in shape was caused by an additional yield. A double yield was observed between the hip and knee in 54.5% of animals (arrows). A shift in plot position after recovery reflects greater extension, as the hip becomes approximately two times more extended than the knee at E3.

To examine coordinated movement between HL joints during locomotion, angle–angle diagrams were constructed by plotting the excursion of one joint against another. Coordination between proximal (hip–knee) or distal (knee–ankle) joints was compared to determine the extent of recovery. Angle–angle diagrams display joint excursion, position of the joints during excursion, and the coordination between joints (Basso et al. 1994). In normal locomotion, a curvilinear shape emerges when one joint moves to a greater extent (more excursion) than the other joint (Fig. 4). Fine motor control is made evident by fractionated movement, or independent control of joints. Fractionation is most clearly demonstrated in E2, where HL joints are required to flex while another extends. Intralimb coordination results when a reproducible and precise curvilinear pattern of movement occurs over multiple step cycles.

After recovery from SCI, coordination between distal (knee and ankle) joints is most impaired. Linear rather than curvilinear paths depict poor fractionated joint movements. The linear pattern during stance results from a lack of E2 or yield phase (Fig. 4). The knee and ankle had equivalent changes in excursion and did not flex or extend in opposition to each other. Proximal coordination between the hip and knee was less impaired but a change in shape and position of the angle–angle plot was apparent (Fig. 4). A second flexion occurred at the knee during E2 (arrow, Fig. 4). A double yield was observed in 55% of animals. Prolonged extension is evident by the rightward and upward shift in position of the post op hip–knee and knee–ankle plots. At E3, the hip becomes approximately two times more extended than the knee, demonstrating greater proximal extension (Fig. 4).

### Joint kinematics and timing of muscle activity

**Figure 5:**
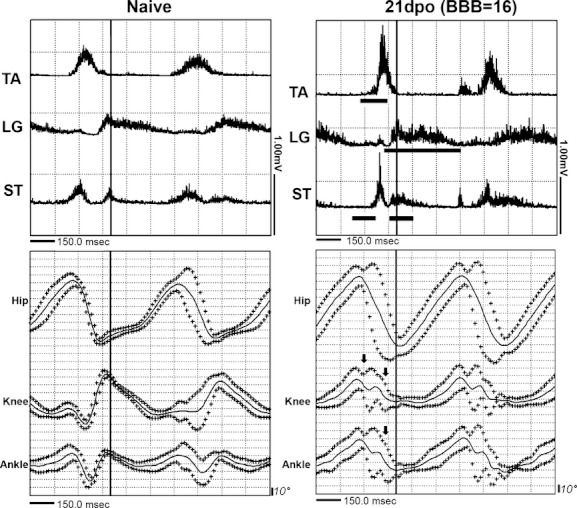
Comparison of HL muscle activity with changes in angular kinematics before and after SCI. EMG activity is aligned with kinematics of the hip, knee, and ankle in the same animal before and 21 days after mild SCI. The vertical line marks stance onset. Black bars represent an overlay of naive burst duration and mean onset relative to ground contact. Before injury, TA onset precedes dorsiflexion while the ankle and knee are still extending. LG precedes plantar flexion prior to ground contact. The double burst of ST aligns with hip and knee extension/flexion prior to initial contact. After recovery from SCI, TA and LG show delayed activation and shorter duration relative to naive. Plantar flexion is absent at the ankle before ground contact. Prolonged dorsiflexion is apparent as TA fires through swing and closer to ground contact. Consequently, LG onset occurs in the absence of plantar flexion and during prolonged dorsiflexion (arrow at ankle). ST2 activation occurs with a second knee flexion before ground contact (arrows at knee) and fires for longer duration after SCI. An absence of E2 in the knee and ankle after SCI may represent variability in step cycles.

In naive animals, TA onset occurs with ankle dorsiflexion while LG onset occurs with plantar flexion before ground contact (Fig. 5). Both muscles are briefly coactive during terminal swing. TA offset occurs prior to plantar flexion and E1 (mean duration = 210.8 msec), and LG remains active during stance (mean duration = 442.9 msec). The dual-burst pattern of ST coincides with extension and flexion in the hip and knee. Onset of ST1 occurs during hip extension (mean duration = 156.8 msec) and ST2 during knee flexion through weight acceptance (mean duration = 248.2 msec). The double burst is separated by a brief pause during E1 while the hip flexes and the knee extends in midswing to move the paw forward.

**Figure 6:**
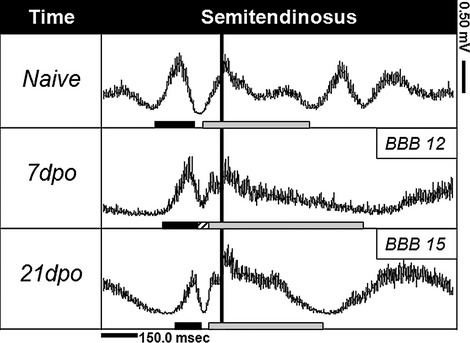
Activation patterns of the semitendinosis change with recovery. EMG activity is plotted in the same animal over time. The vertical line marks stance onset. Seven days after SCI, forelimb–hindlimb coordination and plantar stepping was not consistent (BBB = 12). A loss in a critical reset period between ST1 and ST2 is evident (dashed). ST1 duration (black bar) is noticeably shorter and ST2 is longer (gray bar) after injury. A recovery trend is apparent, but normal bursting does not occur by 21 days. With established coordination and higher stepping frequency in the open field by 21 days, a reset between ST1 and ST2 emerges and ST2 offset is earlier in stance.

Timing and overall pattern of muscle recruitment changed after injury alongside altered joint kinematics. At the ankle, marked changes were evident compared to naive that were maintained throughout recovery. At 21 days, plantar flexion is absent at the ankle and LG onset instead occurs during a period of prolonged dorsiflexion before ground contact (Fig. 5). A reduction in burst duration is apparent in both muscles relative to naive-TA (–25.6 ± 7.5%); LG (–44.1 ± 12.0%). These reductions were independent of recovery in the open field (Fig. 6).

**Figure 7:**
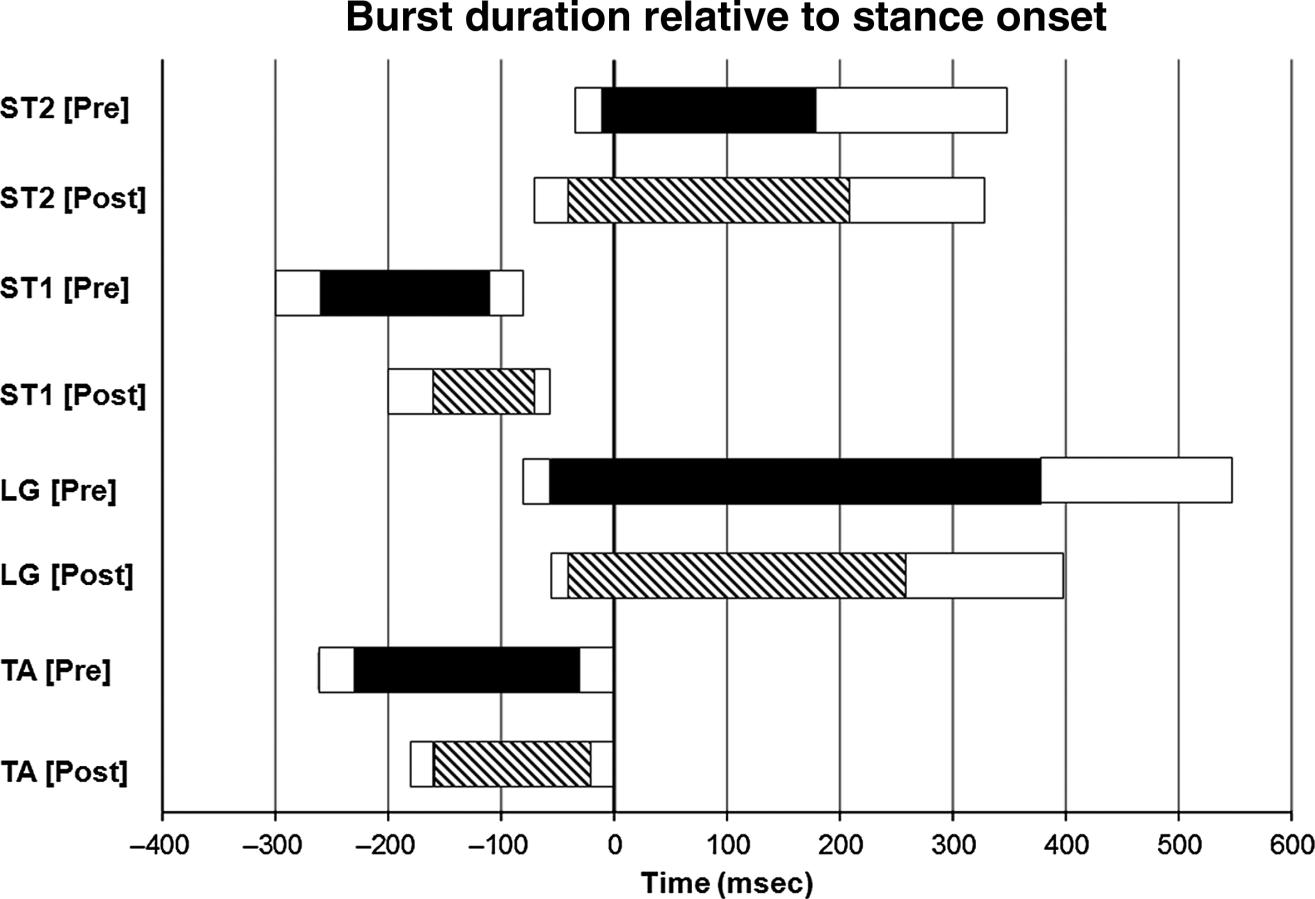
Average burst duration relative to stance onset. Burst durations were measured relative to stance onset (“0”) and averaged before (solid) and 21 days (hatched) after injury. Average EMG onset and offset times are marked by the beginning or end of the shaded regions. Unshaded regions represent the standard deviation of burst onset or offset. TA, LG, and ST1 exhibit shortened burst patterns that occur closer to initial contact after SCI (–25.6 ± 7.5%, –44.1 ± 12.0%, and –11.3 ± 24.5% reduction, respectively). Activity of ST2 shows increased burst duration +33.6 ± 46.13%, with an earlier onset and later offset and marked variability between animals.

**Figure 8:**
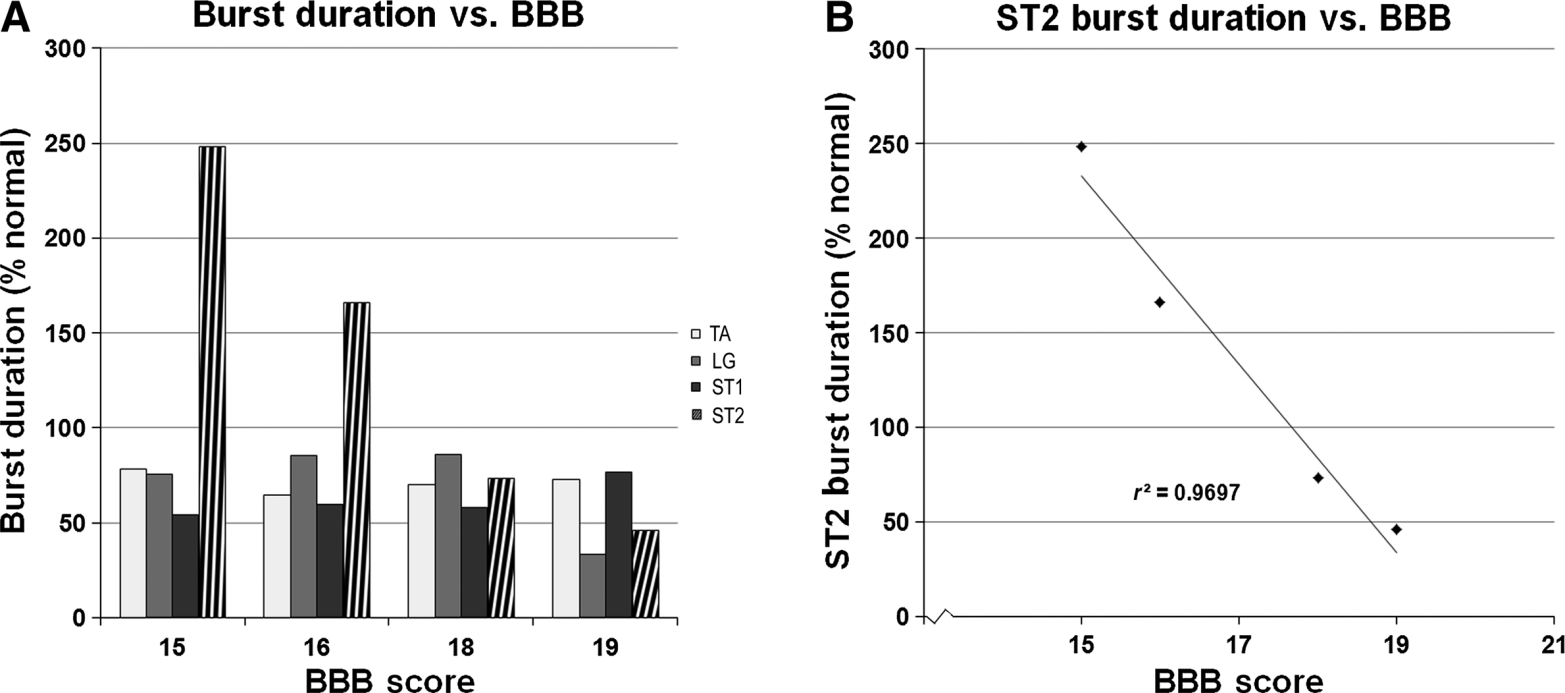
ST2 burst duration predicts recovery in the open field. Normalized burst durations were correlated with BBB scores ranging from 15 to 19. TA, LG, and ST1 display shortened burst durations relative to normal that do not correlate with open field performance (A). Variability observed in ST2 duration correlated (*r*^2^ = 0.9697) with over ground BBB scores (B). Longer bursting in ST2 is associated with greater residual deficits in the open field (*P* < 0.05; significance determined with Pearson's correlation analyses).

Activity of ST changed over time but did not return to normal by 21 days. Early after SCI, with only frequent stepping and limited forelimb–hindlimb coordination (BBB = 12) at 7 days, the dual-burst pattern of the ST is lost and only a single prolonged burst occurs. Dual bursts return by 21 days when coordination and stepping frequency recover (BBB = 15; Fig. 6). ST1 fires later throughout recovery and occurs ∼101.9 msec closer to initial contact, and for shorter duration (–11.3 ± 24.5%) compared to naive (Fig. 7). After recovery, ST2 activation is delayed (35.9 msec) and fires at higher amplitude compared to 7 days. There is notable variability in ST2 firing patterns, as ST2 duration was on average +33.6 ± 46.13% longer at 21 days (Fig. 6). In low (BBB = 16), but not high performing animals, ST2 activation occurs with knee flexion instead of extension during yield (Fig. 5). To determine whether differences in ST2 duration were linear with recovery, burst durations were normalized (percent change postinjury) and correlated with open field BBB scores. A high correlation between ST2 burst duration and BBB scores (*r*^2^ = 0.9697; *P* < 0.05) indicates that smaller changes in burst duration occur in high-performing animals (Fig. 8).

### Changes in ST reflect task specificity

**Figure 9:**
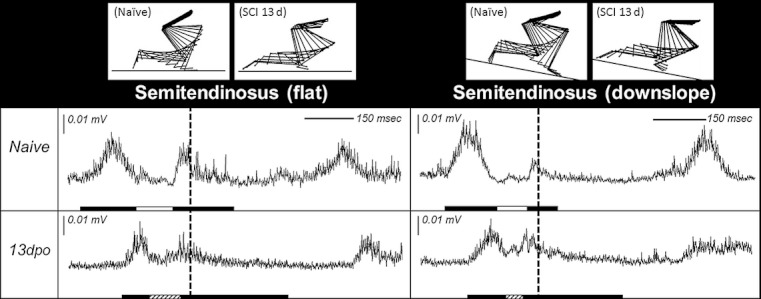
Task-specific changes in locomotion alter ST recruitment after mild SCI. EMG recordings are shown for the same animal as Naive, and 13 days after injury while walking on flat or 10% downhill TM surface grades. Stick figure diagrams at 60 Hz show a representative step cycle from the same animal for each condition. For EMG records, black bars illustrate burst durations during flat or downhill TM walking. White bars represent a defined reset period between ST1 and ST2, while hatched regions identify a nondistinct reset. The dotted line represents peak activation of ST2. Flat TM walking resulted in very little activation of ST2 after SCI, compared to Naive. During downhill walking, a more defined reset period between ST1 and ST2 is evident and ST2 fires at greater amplitude.

To determine whether different forms of TM locomotion alter muscle recruitment after SCI, we compared flat or 10% downslope grade TM walking in the same animals. Similar to 7 days and 21 days, flat TM walking at 13 days showed delayed activation of ST1 and shorter-burst durations relative to normal. During flat walking, a single prolonged burst with an indiscriminate reset period occurs in ST and ST2 is negligible (Fig. 9). TM walking at a downslope grade required a different recruitment pattern that was identified by changes in the ST. Downslope walking produced later, and less activation of TA for ankle dorsiflexion and recruitment of LG was unchanged (data not shown). In the ST, downslope walking re-established a dual-burst pattern (Fig. 9). Notably, ST2 fired at a greater amplitude with a more defined onset/offset period during downslope walking than flat TM walking (Fig. 9). While downslope walking produced a reset period between ST1 and ST2 within the time period described for Naives, the muscle was not silent.

## Discussion

### Overview of the current study

The current work identifies fundamental components of locomotor control that are impaired after recovery from SCI. Despite rapid improvements acutely after injury, deficits persist and normal locomotion does not return by chronic periods. Plateaued walking behavior was characterized by kinematic impairments in yield depicted by a significant decline in angular excursion during the eccentric period of stance (E1–E2). Walking patterns were further characterized by changes in HL muscle recruitment. Delays in activation of knee and ankle muscles occurred during all phases of locomotion. Eccentric actions of the ST (ST2) were notably impaired during yield and significantly correlated with gross open field recovery. Moreover, we found that ST2 activation responds to downslope TM walking after SCI. Our work suggests that the temporal profile of ST serves as a sensitive indicator of gross recovery and that simple changes in locomotor specificity restore its activity.

### Locomotion in the naive rat

To date, few studies have combined EMG and kinematic measures to describe normal locomotion in the rat (Gruner et al. 1980; Gillis and Biewener 2001; Thota et al. 2005). Even fewer have characterized stepping after SCI (Kaegi et al. 2002; Ballermann et al. 2006; Johnson et al. 2012). Muscle recruitment patterns and gait biomechanics for quadrupedal locomotion are better defined in feline models (Buford et al. 1990; Buford and Smith 1990; Basso et al. 1994; Pratt et al. 1996; Smith et al. 1998). Across models, normal gait patterns require eccentric contractions of the hamstrings to slow the HL during the transition from swing to stance (late E1 and E2) and activation of medial and LG to dissipate impact forces and facilitate weight acceptance after ground contact. Our assessment of naive locomotion agrees with work in the rat and cat (Buford et al. 1990; Buford and Smith 1990; Smith et al. 1998; Thota et al. 2005; Fig. 5). We show that peak activation of TA occurs during ankle dorsiflexion and LG during plantarflexion. Similar to what is shown in the cat, a dual-burst pattern of ST occurs during hip and knee movements (Smith et al. 1998). The first burst (ST1) starts before liftoff and continues through peak flexion in swing and is separated from the second burst by a reset period during early E1. The second burst (ST2) decelerates the HL prior to ground contact in late E1 and remains active during the E2 yield phase (Fig. 5).

### Changes in neuromotor control after mild SCI

**Figure 10:**
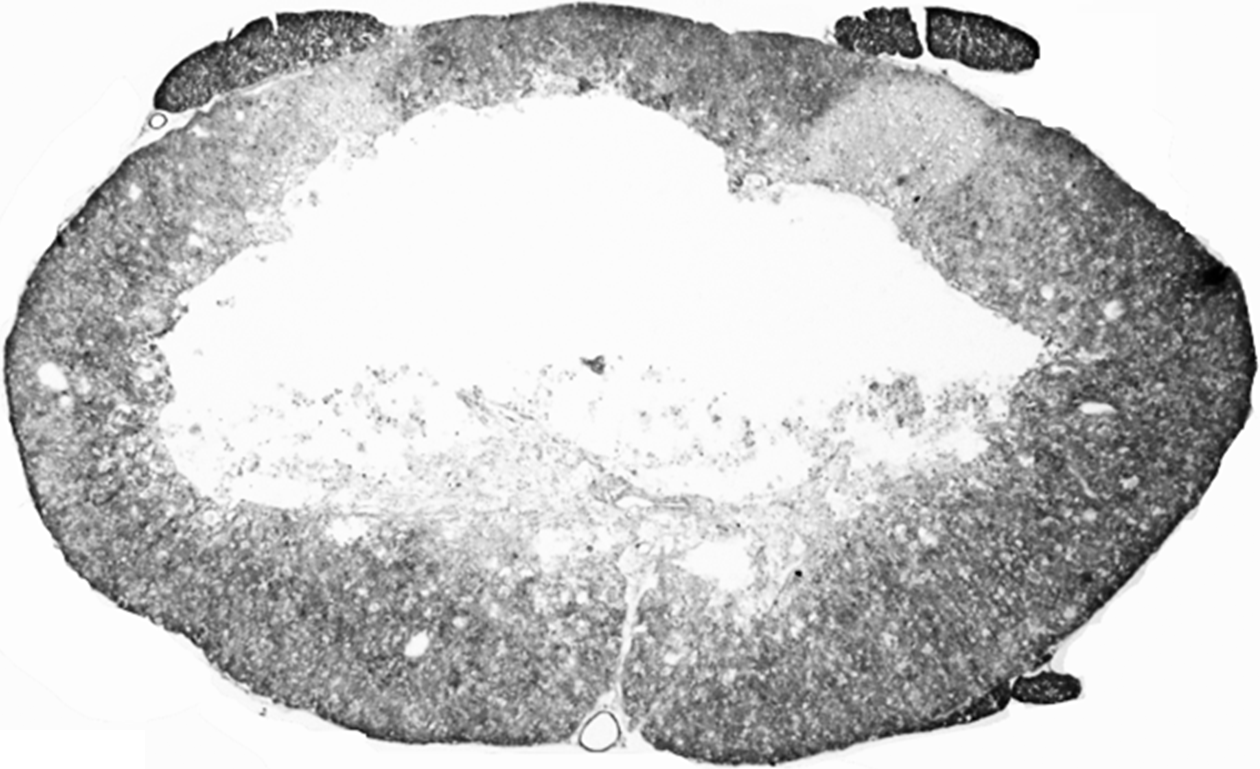
Mild contusion injury results in a central core lesion and peripheral rim of spared white matter. Image depicts the injury epicenter of an animal with a final BBB score of 18 and 64.9% white matter sparing.

**Figure 11:**
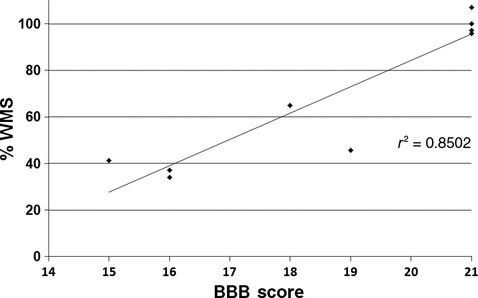
The extent of open field recovery correlates with white matter sparing. Endpoint BBB scores are plotted against the percentage of spared white matter (*r* ^2^ = 0.8502; *P* < 0.01). Significance determined using Pearson's correlation analysis.

Contusive SCI produces distinct neuropathology with a central core lesion and a peripheral rim of spared white matter that replicates clinical SCI (Bunge et al. 1993; Stokes and Jakeman 2002; Fig. 10). Even with partial sparing of ascending and descending systems, the complex cellular sequellae prevents complete locomotor recovery (Basso 2000; Weaver et al. 2002; Detloff et al. 2008; Fig. 11). Previously, we showed that toe dragging, trunk instability, and paw rotation was associated with white matter sparing between 25 and 60% (Kloos et al. 2005). Here, mild contusion with 34–65% sparing not only produced these persistent deficits during open field locomotion but also significant changes in TM kinematics. The new walking pattern included a more caudal limb position during all phases of gait. As a result, joint angular excursions increased from late stance to mid swing (E3, F phases) but decreased from late swing into yield (E1, E2 phases). Thus, it appears that greater excursion is required to overcome the caudal shift in limb position during the propulsive phases of the step cycle. Unlike the cat, greater flexion was not associated with hypermetria as the paw height during swing was normal (Basso et al. 1994; Fig. 2). Interestingly, the locomotor phases with prominent joint deceleration and lengthening contractions had below normal excursions. This reduction in kinematics during E1 and E2 may be due in part to aberrant motor control strategies. Indeed, alterations in fine control of intralimb coordination are prominent during E1 and E2 phases for both proximal and distal joints (Fig. 4). Moreover, a prevalent, almost uniform delay in neural recruitment of distal HL muscles occurred for the TA, LG, and ST1 (+37.1%, +41.04%, +45.1%, respectively; Fig. 7). To our knowledge, we are the first to quantify recruitment latencies after experimental SCI in rats. Contrary to other SCI models, we did not observe increased recruitment of erector spinae musculature (data not shown), nor did we find aberrant coactivation between muscle pairs or across adjacent joints (Ballermann et al. 2006).

### Eccentric motor control is impaired after SCI

Eccentric motor control is a complex skill that emerges late in development (Enoka 1996). During an eccentric contraction, the CNS regulates motor neuron activation to produce muscle forces below an external load resulting in active lengthening. Thus, each lengthening contraction represents the integration of afferent input regarding load and stretch with descending recruitment of motor neurons. Precise CNS modulation prevents muscle spindle-induced stretch reflexes from triggering uncontrolled spasticity after SCI. Other benefits of eccentric contractions include priming the contralateral limb for increased force production, reduced fatigue, and increased metabolic efficiency (Enoka 1996; Grabiner and Owings 1999; Lindstedt et al. 2001). While eccentric actions occur in various parts of the step cycle, the clearest and most predominant occurrence is during weight acceptance or yield phase (E2) when ST and other hamstring muscles lengthen to dissipate impact forces. Our finding that eccentric excursion during yield is markedly impaired across the knee and ankle after contusion confirms previous findings in cats with hemisection SCI (Helgren and Goldberger 1993; Basso et al. 1994; Fig. 4.). It appears that eccentric control of weight acceptance is negatively impacted after SCI and little recovery occurs regardless of injury mechanism or severity.

The ST has a distinct eccentric period of activation that helps determine central pattern generator (CPG)-directed locomotion. Activity in the ST reflects the integration of descending motor drive and afferent input from the limb (Pratt et al. 1996). Phasic sensory signals provided by the second, eccentric burst (ST2) appear to be most important given that it is completely abolished by deafferentation in decerebrate cats and is absent in fictive locomotion unless excitatory drugs are applied (Grillner and Zangger 1984; Grillner and Wallen 1985; Pearson 2004). The magnitude of ST2 activation relates to the rate of knee extension, which suggests that stretch sensitive receptors in ST provide afferent signals to CPGs for locomotion (Wisleder et al. 1990).

We show that recruitment of ST changes over time with recovery. In acute stages, the dual-burst pattern in ST is absent (Fig. 6). A lack in reset between ST1 and ST2 presents a major challenge for a transition to eccentric deceleration in preparation for ground contact. This loss may explain why stepping is not consistent at 7 days. The reset between bursts re-emerges alongside greater activation of ST2 by plateau, but normal patterns are not restored. Interestingly, burst onset and duration of ST2 was the most variable between animals (Fig. 8). Moreover, ST2 activation fails to initiate knee extension before ground contact in low, but not high performing animals (Fig. 5). Thus, it is possible that the integrative function of ST improves with recovery. To determine whether changes in ST were linear with recovery, we compared burst durations of all muscles against open field performance. We found a striking correlation between ST2 duration and BBB scores (Fig. 8). Walking patterns with refined burst duration and a re-established reset period between ST1 and ST2 occurred in animals with greater recovery in the open field. Our work suggests that the temporal profile of ST2 provides a sensitive indication of the spared motor control after SCI. Activity in ST likely reflects the successful integration of spared descending and afferent-driven signals. Facilitating sensorimotor integration in ST may optimize recovery.

### Targeted changes in locomotor specificity restore eccentric control after SCI

Activity in ST reflects task-specific changes in locomotion. In the cat, Buford and colleagues show that recruitment of ST changes between forward and backward walking (Buford et al. 1990; Buford and Smith 1990). Similar to our findings early after SCI (Fig. 6), backwards walking eliminated dual bursting and instead elicited a prolonged single burst. The author suggests that the single ST burst may reflect a generic pattern that is modulated by afferent input to produce a double-burst pattern typical in normal locomotion. Clearly, our findings after SCI suggest a lack of supraspinal control across lumbar CPGs. Whether changes in locomotor specificity facilitate activation across lumbar centers after SCI remains unexplored.

Eccentric actions of the ST are accentuated by changing the grade of the TM belt. Steeper grades of downhill TM walking generate progressively greater activation in both bursts of the ST (Smith et al. 1998). After SCI, we find that downslope walking restores a previously dormant ST2 burst (Fig. 9). In early stages of recovery, we show that flat TM walking produces a single prolonged burst in the ST. By tilting the TM belt to a downslope grade, the same animal at the same point in time produces a completely new motor pattern. Indeed, downslope walking restored a reset period and produced greater and more defined activation of ST2. Thus, the rat retained the capacity to produce controlled ST activation in a task-specific manner. This effect may not be observed after more severe lesions, as feline models show an inability to modulate amplitude with slope changes (Brustein and Rossignol 1998).

## Conclusions, Limitations, and Future Directions

This study identifies essential features of motor control that do not recover after SCI. Impaired eccentric activity during yield is made evident by changes in kinematics and muscle recruitment. Activity in the ST plays a unique role in locomotor integration and reflects task specificity. Here, we show that impaired actions in ST occur with deficits in yield. Furthermore, we show that improvements in ST functionality indicate the extent of recovery. Whether residual impairments may be resolved after SCI by employing targeted tasks that accentuate eccentric control remains unexplored and warrants further investigation. Changes in locomotor specificity would provide a simple adaptation for current clinical practice.

A limitation to our study is that we could not measure relative amplitude of EMG patterns. Because electrodes were implanted to a chronic time period, we expected exact measurements to be unreliable. In same day recordings (i.e., Fig. 9), interpretations of amplitude are more reliable.
